# Feasibility Trial Evaluation of a Peer Volunteering Active Aging Intervention: ACE (Active, Connected, Engaged)

**DOI:** 10.1093/geront/gnz003

**Published:** 2019-02-19

**Authors:** Afroditi Stathi, Janet Withall, Janice L Thompson, Mark G Davis, Selena Gray, Jolanthe De Koning, Graham Parkhurst, Liz Lloyd, Colin Greaves, Robert Laventure, Kenneth R Fox

**Affiliations:** 1 School of Sport, Exercise and Rehabilitation Sciences, University of Birmingham, UK; 2 Department for Health, University of Bath, UK; 3 Physical Activity Measurement Consultant, Bwlch, Wales, UK; 4 Faculty of Health and Applied Sciences, University of the West of England, Bristol, UK; 5 Centre for Transport and Society, Department of Geography and Environmental Management, University of the West of England, Bristol, UK; 6 School of Policy Studies, University of Bristol, UK; 7 Later Life Training LTD, Perthshire, UK

**Keywords:** Physical activity, Physical function, Community engagement, Intervention, Peer support, Mixed methods, Process evaluation

## Abstract

**Background:**

ACE (Active, Connected, Engaged) is a theory-informed, pragmatic intervention using peer volunteering support to promote active ageing in socially disengaged, inactive older adults. This study aimed to establish ACE’s feasibility and acceptability.

**Methods:**

Fifty-four older adults were recruited as either peer volunteers (activators; *n* = 15) or participants (ACEs; *n* = 39). Participants were randomized to one-to-one support from an activator (ACEs-Intervention [ACEs-I]) or a waiting-list control group (ACEs-Control [ACEs-C]). Activators supported ACEs-I to get out more and engage with local activities. Objectively measured physical activity (PA), lower limb function, and number of out of house activities were assessed at baseline and post-intervention. A mixed-methods process evaluation assessed changes in confidence to get out and about, social support, autonomy, competence, and relatedness.

**Results:**

Eighty-two percent of ACEs (mean age = 73.7 years [*SD* 7.3]) and all activators completed assessments at both baseline and post-intervention (6 months). ACEs-I reported more out of house activities (*M* [*SD*] = 6.34 [4.15]). ACEs-I increased physical function post-intervention (*M* [*SD*] = 9.8 [2.3]). ACEs-I reported improved well-being and vitality and increased confidence to get out and about, confidence in the face of specific barriers, knowledge of local initiatives, and perceived social support post-intervention. Activators, although sufficiently active at baseline, increased their PA further. ACE was well-accepted and easy to deliver.

**Conclusions:**

ACE is an acceptable and feasible intervention for helping socially disengaged older people to get out and about more, improve their confidence, and engage more with their community.

Few older people achieve the recommended 150 min of moderate-to-vigorous-intensity physical activity (MVPA) each week (Keadle, McKinnon, Graubard, & Troiano, 2016). The greatest gains in life expectancy and life quality are likely to occur by moving from the current very low levels of PA to some activity. Lee and coworkers (2012) reported that inactive people would gain 1.3–3.7 years from age 50 years by becoming active.

The validity of the simple “just get out of the door!” active ageing message (Simonsick, Guralnik, Volpato, Balfour, and Fried, 2005) is supported by the Older People and Active Living (OPAL) observational and longitudinal studies (Fox et al., 2011, 2015; Simmonds et al., 2014). These studies demonstrated that number of daily trips and steps, done for any reason, using any form of transport or walking, were associated with positive physical and mental health profiles in people aged 70 and older (Davis et al., 2011; Withall et al., 2014). The importance of getting out and about is further supported by the strong association between lower life-space mobility (defined as the size of the spatial area in which a person moves in everyday life, the frequency of going out, and the need for assistance) and cognitive impairment (James, Boyle, Buchman, Barnes, & Bennett, 2011), frailty, poor physical functional health, disability, and mortality (Kennedy et al., 2017).

Social connectedness is as influential on survival as recognized health risks such as smoking and obesity (Holt-Lunstad, Smith, Baker, Harris, & Stephenson, 2015). The Scottish longitudinal cohort study identified social connectedness and health status as independent predictors of change in PA in community-dwelling people aged 65 years and older (Clarke et al., 2017). In addition to physical gains, getting out and about on a daily basis generates more opportunities to engage with meaningful pursuits and connect socially, and boost perceptions of autonomy and competence, enhancing social, emotional, and physical well-being (Polku et al., 2015).

Several personal, social, and environmental barriers discourage older people from leaving their home regularly (de Koning, Stathi, & Fox, 2015). Perceptions of poor health, not having “a reason to leave home” and ‘no-one to go out with,” have been identified as high-impact barriers (Stathi et al., 2012). As people get older they are at increasing risk of a downward spiral of reduced activity and less social engagement, resulting in decreased physical capacity and compromised health (Shankar, McMunn, Demakakos, Hamer, & Steptoe, 2017). Finding ways to break this downward spiral of inactivity and disengagement is a major public health challenge.

Enlisting peer volunteering support has potential to be an effective strategy for increasing PA in older adults, particularly those who are very inactive and socially disengaged. Volunteering is growing in popularity among older people and tends to remain a prominent behavior until at least middle-old age (mid-70s) (McMunn, Nazroo, Wahrendorf, Breeze, & Zaninotto, 2009). Evidence supports the contribution of volunteering to social connectedness (Parkinson, Warburton, Sibbritt, & Byles, 2010), mental well-being, quality of life, self-esteem, active lifestyle, and delayed mortality (Cattan, Hogg, & Hardill, 2011; McDonnall, 2011; McMunn et al., 2009; Wahrendorf & Siegrist, 2010). Peer volunteers act as positive role models who support and empower older people, and peer-led approaches have potential to be cost-effective and sustainable (Allen et al., 2016; Peel & Warburton, 2009).

In order to provide robust evidence of the efficacy of this approach, we systematically developed a theory-informed, low-cost, pragmatic intervention (ACE: Active, Connected, Engaged), using peer volunteers (activators) to support participants to get out more and engage with initiatives in their local communities (Withall et al., 2018). We employed the Process Model of Lifestyle Behaviour Change (PMLBC; Greaves, 2012) which was developed from a wide-ranging systematic review of evidence of components associated with success in interventions to change diet and/or PA (Greaves et al., 2011). The model has been used in several lifestyle change interventions that have been subject to trial evaluations (Greaves et al., 2015).

We hypothesized that the recipients of the ACE intervention would report more out-of-house activities and better motivation to adopt an active lifestyle in the long term. In this paper, we report the design of the ACE intervention and describe the findings of the feasibility trial. The specific aims of ACE were to:

1. Assess the feasibility of recruitment and retention of activators and participants to a peer volunteering active ageing intervention.
2. Estimate the potential effect of the intervention on engagement in activities outside the home, PA, and physical function.
3. Assess the applicability of the theoretical framework adopted for this intervention.
4. Examine the acceptability of the intervention and trial methods to participants and activators.

## Methods

A two-arm randomized control feasibility trial was used to evaluate the ACE intervention.

### Recruitment

Participants were recruited from two wards in Bristol, United Kingdom chosen from a range of 14 neighborhood partnerships. These wards were selected to provide diversity based on several criteria including percentage of older residents, level of deprivation, and extent of provision of leisure facilities for older adults. This information was gathered via: (a) statistical profiles from Bristol City Council (http://www.bristol.gov.uk/page/council-and-democracy/neighbourhood-partnership-statistical-profiles) and (b) the Mosaic Public Sector system which provides details of U.K. citizens’ location, demographics, lifestyles, and behaviors (http://www.experian.co.uk/public-sector/index.html). The study was approved by the University of Bath REACH ethics committee (EP 11/1298). Participants were informed about the study in writing and in person, and provided written informed consent.

Participants included: (a) the *ACEs*: These were sedentary retired adults aged 65 and older who reported spending less than 20 min per week in the past month in MVPA, were capable of walking at least 200 m, and did not have a diagnosis of dementia. Inclusivity was emphasized but we excluded people with disease or disability that seriously precluded participation in out-of-house activities, people who were already meeting current PA recommendations, and people who were regularly engaging with local groups and activities; (b) the *activators* (the volunteers). Using established communication channels within the two neighborhood partnerships and via the recruitment mailing described below, we recruited adults aged 60 and older to act as peer volunteers supporting intervention participants (ACEs-I); and (c) two part-time *coordinators.* One part-time (0.2FTE) coordinator in each ward was employed by the University of Bath but had no research role. Their tasks were to form working relationships with community groups, coordinate activator training sessions, support activators in the intervention delivery, and represent ACE locally.

Participants were recruited via a letter of invitation. Based on our formative research (Stathi, Fox, Withall, Bentley, & Thompson, 2014; Withall et al., 2018), the recruitment materials focused on provision of assistance with getting out and about and engaging within communities. A commercial mailing list of residents in the relevant postcodes who were 65 and older was purchased (£510), and approximately 2,000 invitation letters and reply slips were mailed (£1,100). An A5 flyer (148 mm × 210 mm) highlighting key messages was also delivered to 1,000 homes in one of the intervention areas (£420). Posters were displayed in local health centers, libraries, and community centers, and local community groups and professionals were asked to refer potential participants. People interested in taking part were sent an information pack, a reply form requesting basic demographic information, and a reply-paid envelope (£55). Once screened and deemed eligible, ACEs were randomized to either the intervention [ACEs-I] or the control [ACEs-C] arm by choosing an envelope containing random allocation information. ACEs-I were then paired with an activator based on shared interests and geographical proximity of their residences. Given the gender imbalance between activators and ACEs-I, some pairings were necessarily a gender discordant match. No pairings were actively rejected by either party for this reason.

The invitation letters included an option for people to express an interest in the activator role. Those who did were invited for interview and if deemed eligible, were invited to the baseline assessments where they could meet the ACEs-I. Eligibility criteria were being 60 years old or over, physically capable of getting out and about locally, good communication skills, two references, and a successful Criminal Records Bureau (CRB) check.

### Intervention Development and Delivery

The development of the ACE intervention was one of the outcomes of a collaborative network, AVONet, which used a range of approaches to identify best bet active ageing promotion strategies (Stathi et al., 2014).

**Figure 1.**
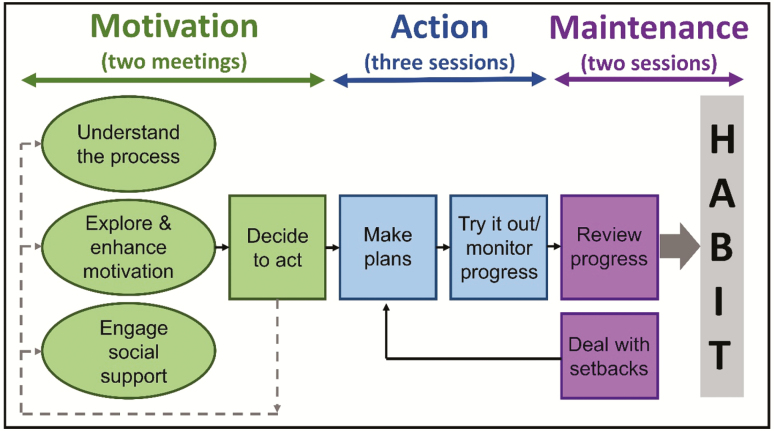
The Process Model of Lifestyle Behaviour Change, an adapted version of the Health Action Process model (Greaves, Reddy, & Sheppard, 2010).

During the first 9 months of the 24-month ACE study, feedback on the content and delivery of the intervention and the proposed behavioral strategies was sought during extensive participant and public engagement activities including a series of focus groups and one-to-one interviews (Withall et al., 2018). All proposed changes were mapped onto the PMLBC, which was used to map out the intended processes of behavior change during the three stages of the ACE intervention: motivation, action, and maintenance (see Figure 1; Greaves, 2012). The principles of Self-Determination Theory (Ryan & Deci, 2000) influenced the development of the intervention content and training of the activators to help them facilitate the development of ACEs-I’s autonomous motivation, confidence, and competence for getting out and about. Activators attended a 2-day training course and received a manual to support the training and as a reference tool during the intervention delivery. Both the training and manual contained a protocol for type and frequency of interactions with the ACEs-I but some flexibility was encouraged depending on individual needs.

### Description of Intervention and Control Groups

ACEs-I were invited to attend a 6-month program. This included two one-to-one initial meetings with their activators to support motivation (Motivation stage – first 2 weeks), to enable ACEs-I and activators to get to know each other, to review potential local activities, and to consider and address any barriers to participation. At least three joint visits to local initiatives of the ACEs-I’s choice followed (Action stage – month 1–3). Further support to continue attending local activities was provided by telephone and at least two further joint visits, with the aim of ACEs-I beginning to attend these activities independently during the maintenance stage (month 3–6). ACEs-I engaged with a wide range of activities, physical and non-physical, at both Action and Maintenance stage. These included bowling, ballroom dancing, lunch clubs, tai-chi, walking groups, art classes, yoga, skittles (a game similar to bowling), and special events such as festivals and charity fairs. Two ACE social events were scheduled for all ACEs-I and activators to support exchange of information about local opportunities, celebration of achievements, and facilitation of within-group support for more local engagement. Other than written materials about local initiatives, ACEs-C received no additional input during the period of the intervention, but were offered the intervention after study completion.

### Baseline and Follow-up Measures

As this was a feasibility trial, the main measures of interest were recruitment rate, study completion rate (the proportion of participants providing data at 6 months), and acceptability of the intervention (explored via interviews and focus groups). Intervention attendance (mean number of the sessions attended from the seven sessions scheduled for ACEs-I and their activators) was also measured.

Process evaluation was conducted to determine the relative usefulness of different intervention components and identify ways to refine/improve the intervention (Moore et al., 2015). This included:

a) *Quantitative process evaluation* via a self-administered questionnaire which assessed changes in the motivation variables targeted by the PMLBC model (importance, self-efficacy, social support) and the three basic needs (autonomy, competence, relatedness) identified within Self-Determination Theory and measured by the Basic Needs Satisfaction-General Scale (BNS-GS) (Gagné, 2003).
b) *Qualitative process evaluation* via semi-structured exit interviews and focus groups with a selection of 20 ACEs-I, 13 activators, and 2 coordinators, to evaluate which elements of the intervention worked well and which could be improved. Due to space constraints, a brief overview of key areas for improvement is presented in the “Results” section and in Supplementary Appendix A.

Several outcome measures were also included in the assessment protocol to identify those most appropriate for inclusion in a future definitive trial together with demographic data (i.e., age, gender, marital status, ethnicity, education level reached, type of residence, house ownership status, and years at current address).

#### Physical activity

PA included: (a) Journeys outside home: ACE participants and activators completed daily journey logs for 7 days recording the time, purpose, and mode of transport (e.g., walking, cycling, driving, car passenger, bus, train, or “other”) for each journey, similar to that used in previous studies (Davis et al., 2011; Fox et al., 2011). Data were reduced to provide a mean number of daily journeys; and (b) Accelerometry: ACE participants and activators were asked to wear a Actigraph GT3X accelerometer for 7 days during waking hours removing it only when bathing. It was worn in a pouch attached to the participant’s belt or a provided elastic belt. Data inclusion criteria were as tolerant as possible to accommodate variable compliance among participants; at least 8 hr of data on at least 1 day was required. Data reduction was performed using Actigraph software ActiLife v 4.4.1 Firmware v6.0.1 to derive: Sedentary time (<100 counts per minute [CPM]), Light PA (100–759 CPM), Lifestyle PA (760–1,951 CPM), and Moderate-to-Vigorous PA (MVPA; >1,952 CPM) (Ward et al., 2005). We assessed Lifestyle PA (including, but not limited to, casual walking, stretching, light weight training, dancing slowly, and light house/garden work) as these are typical activities of this population and there is increasing evidence for their health benefit (Loprinzi, Lee, & Cardinal, 2015).

#### Lower limb function

ACEs’ lower limb function was assessed using the Short Physical Performance Battery (SPPB). The SPPB measures lower extremity function using activities that mimic tasks key to maintaining independent living: static balance (three balances), gait speed (4-m walk), and leg strength (getting up and down from chair) (Guralnik et al., 1994).

#### Well-being

An interviewer-administered questionnaire included: (a) four well-being items from the Well-being Annual Population Survey which assesses global perceptions of life satisfaction and levels of happiness and stress in the previous day (Self, Thomas, & Randall, 2012); (b) the Ageing Well Profile (AWP), a multi-scale measure of subjective well-being providing estimates of social (AWP Social), physical (AWP Physical), mental (AWP Mental), and developmental (AWP Developmental) well-being (Stathi & Fox, 2004); (c) five subscales of the Resilience Scale (Meaningful life, Perseverance, Self-reliance, Equanimity, and Existential aloneness) (Wagnild & Collins, 2009); and (d) the six-item Vitality Scale (Trait) (Bostic, Rubio, & Hood, 2000).

#### Activity barriers

Fourteen items assessing perceived personal, social, and environmental barriers to neighborhood activity (European Commission—Pan-EU Survey, 1999) were included. All baseline assessments took place after screening and before randomization. Participants were asked to complete the questionnaires, perform the SPPB tests, and were administered the accelerometer with a manual of how to use it for the next 7 days. After completion of the intervention, all participants were asked to repeat the same protocol.

Baseline and 6-month data, described above, were collected in a 1.5- to 2.5-hr session either at the activator’s or participant’s home or a local community center. Accelerometers and the PA logs were returned after 7 days in a reply-paid envelope.

### Data Analysis

Descriptive statistics (frequencies, percentages, means, *SD*s, 95% confidence intervals [CIs]) were generated to describe the recruitment and attendance data. To test for pre–post changes in PA variables between baseline and 6 months, paired samples *t*-tests were conducted separately for the control, intervention, and activator groups. As this was a feasibility trial, the sample size was not powered to detect differences either within or between groups. Hence, 95% CIs around estimates of mean differences are presented, rather than *p*-values are presented for ACEs-I, ACEs-C, and activators. Principles of framework analyses were employed to analyze focus groups and individual interviews (Ritchie and Lewis, 2003).

## Results

### Participants

Fifty-four older adults were recruited as ACE participants (*n* = 39) or activators (*n* = 15). Table 1 presents the sample characteristics; 82% (95% CI: 70–94) of ACE participants provided quantitative data at both baseline and 6 months.

**Table 1.** Participant Characteristics

	ACEs-I (*n* = 22)	ACEs-C (*n* = 17)	Activators (*n* = 15)
	*n* (%)	*n* (%)	*n* (%)
Gender (M/F)	12/10	10/7	2/13
Age, mean (*SD*)	72.9 (7.3)	75 (6.4)	68.7 (4.4)
Marital status			
Widowed	8 (36.4)	6 (35.3)	5 (33.3)
Married	7 (31.8)	8 (47.1)	3 (20.0)
Single/divorced	7 (31.8)	3 (17.6)	7 (46.7)
Ethnicity			
White Caucasian	21 (95.5)	17 (100.0)	15 (100.0)
Other	1 (4.5)	0 (0.0)	0 (0.0)
Highest education level completed			
Primary school	0 (0.0)	0 (0.0)	0 (0.0)
Middle school	0 (0.0)	1 (5.9)	0 (0.0)
Some secondary school	0 (0.0)	0 (0.0)	0 (0.0)
Completed secondary school	15 (68.2)	9 (52.9)	5 (33.3)
Some college or vocational training	2 (9.1)	2 (11.8)	3 (20.0)
Completed college or university	4 (18.2	4 (23.5)	5 (33.3)
Completed graduate degree or higher	1 (4.5)	1 (5.9)	2 (13.3)
Home ownership status			
Own	18 (81.1)	15 (88.2)	14 (93.3)
Renting	4 (18.2)	2 (11.8)	1 (6.7)
Type of residence			
Bungalow	2 (9.1)	2 (11.8)	1 (6.7)
Terrace house	3 (13.6)	1 (5.9	5 (33.3)
Semi-detached house	11 (50.0)	8 (47.1)	5 (33.3)
Flat	3 (13.6)	1 (5.9)	1 (6.7)
Detached house	2 (9.1)	3 (17.6)	3 (20.0)
Sheltered housing	0 (0.0)	1 (5.9)	0 (0.0)
Other	1 (4.5)	1 (5.9)	0 (0.0)
Years at current address, mean (*SD*)	23(12.9)	30(13.7)	21.8(17.1)
Sedentary, min/day	681.5 (74.9)	616.2 (112.3)	658.9 (66.8)
Light, min/day	102.0 (37.8)	115.8 (26.0)	106.7 (16.5)
Lifestyle, min/day	36.8 (16.3)	65.9 (18.2)	51.9 (24.0)
MVPA, min/day	17.7 (13.2)	34.5 (6.7)	39.1 (25.6)
Steps/day	3,714 (1,616)	6,242 (1,549)	6,289 (3,340)

*Note:* ACEs-C = ACEs-Control; ACEs-I = ACEs-Intervention; MVPA = moderate-to-vigorous-intensity physical activity.

### Aim 1. Feasibility of Recruitment and Retention

Two thousand mailed invitations and 1,000 flyers were delivered in the target areas resulting in 230 responses from potential participants and activators (response rate 11.5% [95% CI: 10.1–12.8]). Limited response rate data are available from similar studies for comparison purposes. The Groningen Active Living Model study completed baseline measures on 315 older adults having mailed 8,504 people (3.7%). Recruitment rates were supported by reminders and home visits for potential participants. Retention figures were not reported (Stevens, de Jong, & Lemmink, 2008).

#### ACE participant

We received 154 requests for information packs. Sixty-five people returned reply forms. Twelve people were screened out due to high activity levels. Three people did not respond to further phone calls and messages. Forty people consented to take part. One person died prior to baseline measures; therefore, the baseline sample includes 39 people. Seven people dropped out prior to final measures due to ill-health (*n* = 3), carer commitments (*n* = 2), lack of time (*n* = 1), and moving to a different city (*n* = 1). The ACE participants were represented similarly across gender and marital status with a mean age (*SD*) of 73.8 (7.3) years (see Table 1).

#### Activators

We received 76 requests for information packs. Twenty people returned their reply forms. One person was screened out for being in full-time (F/T) employment and another went back to F/T employment prior to baseline measures. One person dropped out prior to training (going back to F/T employment) and two people dropped out during/after training (one going back to part-time [P/T] employment, one unwilling to commit to the program schedule). Activators were two men and 13 women, with a mean age (*SD*) of 68.7 (4.4) years. All 15 of the remaining activators completed the ACE program and all provided data at both baseline and 6 months. Eight activators supported one ACEs-I participant each. Seven activators agreed to support two ACEs-I participants each. These activators delivered the intervention with each of their allocated ACEs-I participants separately.

### Aim 2. Changes in Activity, Physical Function, and Well-being

#### Activity and physical function

ACEs-I reported more activities outside the home at (6-month) follow-up (*M* = 6.34; *SD* = 4.15) than at baseline (*M* = 4.39; *SD* = 3.75). ACEs-C did not report changes in the number of activities outside home at follow-up. Both ACEs-I and ACEs-C increased the number of activities done at home.

The baseline characteristics of the sample are reported in Table 1. At baseline, ACEs-I were substantially less active than ACEs-C for minutes of lifestyle activity per day, MVPA, and steps per day and spent more time (55 min per day) being sedentary. Changes in PA from baseline to follow-up for ACEs are presented in Table 2. ACEs-I increased lifestyle PA, MVPA, sedentary time but decreased light activity. ACEs-C increased their light and lifestyle activity, MVPA, and step counts while reducing their sedentary time over the intervention period (Table 2). However, only 8 out of 17 ACEs-C provided us with valid accelerometry data, so their results should be treated with caution as they might not be representative of the group as a whole.

**Table 2.** Changes in Physical Activity Between Baseline and 6 Months

	ACEs-C (*n* = 8)		ACEs-I (*n* = 17)		Activators (*n* = 13)	
	Difference^a^		Difference^a^		Difference^a^	
	Mean (*SD*)	95% CI	Mean (*SD*)	95% CI	Mean (*SD*)	95% CI
Sedentary, min/d	−8.7 (70.7)	−57.6 to 75.1	13.1 (77.2)	−26.6 to 52.8	−30.4 (75.2)	−75.9 to 15.0
Light, min/day	6.7 (19.0)	−9.2 to 22.6	−1.1 (20.2)	−11.5 to 9.3	10.3 (32.5)	−9.3 to 29.9
Lifestyle, min/day	5.1 (17.3)	−9.4 to 19.6	3.9 (11.3)	−1.9 to 9.7	2.9 (22.8)	−10.8 to 16.7
MVPA, min/day	5.6 (15.6)	−7.4 to 18.7	1.0 (8.5)	−3.3 to 5.4	5.5 (25.4)	−9.9 to 20.9
Steps/day	908 (1,687)	−503 to 2,319	14 (1,043)	−522 to 550	926 (2,755)	−739 to 2,590

*Note:* ACEs-C = ACEs-Control; ACEs-I = ACEs-Intervention; MVPA = moderate-to-vigorous-intensity physical activity.

^a^Positive changes represent an increase.

Activators increased their light and lifestyle activity, MVPA, and step counts while reducing their sedentary time over the intervention period (Table 2).

A mean difference of 0.98 points in physical function (95% CI: −0.38 to 2.3) between ACEs-I and ACEs-C groups was reported post-intervention. A difference in change scores of 0.5 is deemed to be a clinically significant difference therefore it is plausible that a definitive trial might find a clinically meaningful difference between the two groups (Table 3).

**Table 3.** Changes in Lower Limb Physical Function (SPPB) Score Between Baseline and 6 Months

	Mean (±*SD*)			
	Baseline	Follow-up	Mean difference	95% CI
ACEs-C (*n* = 9)	10.6 (1.0)	9.6 (1.8)	−1.0 (1.4)	−2.0 to 0.1
ACEs-I (*n* = 22)	9.5 (2.4)	9.8 (2.3)	0.3 (1.8)	−0.5 to 1.1

*Note:* ACEs-C = ACEs-Control; ACEs-I = ACEs-Intervention; CI = confidence interval.

#### Well-being

ACEs reported higher levels of vitality at follow-up (Table 4). ACEs-I reported increased life satisfaction and resilience and better perceptions of physical, mental, and social well-being. ACEs-C reported small increases in mental and social well-being and lower levels of physical well-being and vitality.

**Table 4.** Changes in Well-being Variables Between Baseline and 6 Months

	ACEs-C (*n* = 7–10)			ACEs-I (*n* = 20)		
		0–6-month change^a^			0–6-month change^a^	
	Mean (*SD*) baseline score	Mean	95% CI	Mean (*SD*) baseline score	Mean	95% CI
Developmental well-being	4.00 (0.77)	0.05	−0.39 to 0.50	3.79 (1.09)	0.05	−0.30 to 0.40
Physical well-being	3.56 (0.97)	−0.23	−1.00 to 0.54	3.09 (0.84)	0.12	−0.27 to 0.50
Mental well-being	3.86 (0.96)	0.20	−0.72 to 1.11	3.76 (0.75)	0.14	−0.14 to 0.41
Social well-being	3.65 (0.81)	0.08	−0.59 to 0.75	2.80 (1.03)	0.54	0.12 to 0.96
Resilience	6.28 (0.73)	0.09	−0.43 to 0.61	5.84 (0.94)	0.43	0.22 to 0.64
Vitality	5.04 (1.68)	−0,96	−1.83 to −0.09	4.07 (1.52)	0.72	−0.36 to 1.48
Life satisfaction	7.30 (2.26)	0.80	−0.50 to 2.10	6.09 (2.09)	0.77	0.15 to 1.40

*Note:* ACEs-C = ACEs-Control; ACEs-I = ACEs-Intervention; CI = confidence interval.

^a^Higher scores denote improvement.

### Aim 3. Motivational Processes

The importance of getting out and about was high for ACE participants at baseline and it further increased at follow-up (Table 4). The confidence to get out and about, a key target of change in ACE, increased for ACEs-I as well at their planning ability over the 6 months of intervention. ACEs-C reported lower levels of importance and confidence. Perceptions of social support were higher at follow-up for the ACEs-I but not for ACEs-C (Table 5).

**Table 5.** Changes in Motivational Processes Between Baseline and 6 Months

	ACEs-C (*N* = 7–9)			ACEs-I (*N* = 18–22)		
		0–6-month change^a^			0–6-month change^a^	
	Mean (*SD*) baseline score	Mean	95% CI	Mean (*SD*) baseline score	Mean	95% CI
Importance of getting out and about	9.43 (0.98)	−0.14	−0.78 to 0.50	7.36 (2.4)	0.55	−0.61 to 1.70
Confidence to get out and about	8.77 (1.26)	−0.36	−1.10 to 0.38	7.14 (2.38)	0.13	−0.66 to 0.93
Social support by:						
Encouragement	3.13 (1.64)	0.00	−1.00 to 1.00	2.38 (1.20)	0.81	0.16 to 1.46
Discussing local opportunities	2.50 (1.31)	−0.13	−1.17 to 0.92	1.95 (1.29)	1.00	0.30 to 1.70
Providing information about local initiatives	2.13 (0.99)	−0.25	−1.32 to 0.82	1.67 (1.02)	1.48	0.87 to 2.08
Joining when getting out and about	2.63 (1.69	0.13	−1.25 to 1,50	2.45 (1.44)	0.73	−0.02 to 1.48
Planning to get out and about	3.43 (0.33)	−0.07	−0.65 to 0.50	2.85 (0.68)	0.42	0.05 to 0.78
Competence	4.55 (1.33)	0.29	−0.87 to 1.44	4.13 (1.05)	0.96	0.40 to 1.52
Autonomy	5.71 (0.95)	0.47	−0.34 to 1.28	5.50 (0.85)	0.55	0.19 to 0.91
Relatedness	6.03 (0.63)	0.06	−0.27 to 0.40	5.70 (0.68)	0.12	−0.05 to 0.29
Barriers to get out and about^a^:						
Personal barriers	2.47 (1.49)	−0.14	−1.63 to 1.34	3.49 (1/81)	−0.42	−0.97 to 0.12
Environmental barriers	2.39 (1.67)	−1.58	−1.94 to 0.49	2.88 (2.24)	−0.30	−0.79 to 0.19
Social barriers	4.00 (3.81)	−0.11	−1.52 to 1.30	7.42 (3.56)	−2.89	−4.72 to −1.07
All barriers	2.70 (1.44)	−0.26	−1.43 to 0.91	3.62 (1.56)	−0.51	−1.00 to −0.02

*Note:* ACEs-C = ACEs-Control; ACEs-I = ACEs-Intervention; CI = confidence interval.

^a^Lower scores denote improvement in perceived barriers.

Perceptions of competence and autonomy to get out and about at 6 months were higher at follow-up in the ACEs-I and there was also a decrease in the importance of barriers to get out and about. The lower rating of barriers’ importance and the higher levels of confidence and resilience draw a favorable motivational picture for ACEs-I at 6 months. These changes were evident at follow-up, even though the face-to-face support provided by the activators was reduced gradually in the final 3 months (maintenance stage).

### Aim 4. Acceptability of the Intervention and Trial Methods to ACEs and Intervention Providers

Fourteen semi-structured exit interviews and seven focus groups examining issues related to acceptability of the ACE intervention and trial methods were conducted with 20 ACEs-I, 13 activators, and the two coordinators.

#### Acceptability of ACE intervention

##### Attendance.

All ACEs-I who completed the intervention engaged with their activator at least seven times as planned during the motivation, action, and maintenance stages. However, most ACEs-I engaged more often, especially in the maintenance stage. Of the three ACEs-I who dropped out, two met their activator less than five times but were contacted regularly by phone. Their reasons for drop out (ill-health, carer responsibilities) had already affected their engagement with the program in the early motivation stage.

##### Activators and activities.

ACEs-I rated highly the role of the activators in informing them about local initiatives and facilitating their re-engagement with their community:

It got me back into things, as I said, like painting and…the singing, I probably wouldn’t have thought about that, but I really enjoy it. It’s a fun thing. I certainly wouldn’t have done it if I didn’t get in touch and start coming to ACE. (ACEs-I, Group 1)

The ACE days were well received. All ACEs-I were invited to these two events which included interactive sessions, information sharing and opportunities for socializing:

I think what it did, it made all of the participants realize there were other people like them, and they enjoyed chatting with each other. I think that was part of making them feel like they were part of a group.(Activator, Group 2)

##### Coordinators’ support.

All activators praised the coordinators, highlighting the benefits of the support they provided:

She [the coordinator] was brilliant, she’d ring up regular and say “How did you get on?” she was really nice. (Activator, Group 1)

Supplementary Appendix A provides a list of specific areas for improvement for intervention delivery and research design, including illustrative quotes.

## Discussion

This study examined the feasibility and acceptability of a theory-informed, pragmatic intervention using peer volunteering support to promote active ageing. The ACE intervention was the outcome of a rigorous developmental process during which a range of stakeholders co-produced a program designed to be acceptable, feasible, and make a positive contribution to older people’s health and well-being. The extensive patient and public involvement (PPI) throughout development may have helped produce a community intervention which was well accepted and easy to administer (Stathi et al., 2014; Withall et al., 2018). The importance of extensive PPI is now widely acknowledged, and is an established goal of science policy and a requirement of research funders in the UK (http://www.rds.nihr.ac.uk/wp-content/uploads/RDS-PPI-Handbook-2014-v8-FINAL.pdf).

ACEs included similar numbers of men and women. This is significant as older men are less likely to become involved in activity interventions (Waters, Galichet, Owen, & Eakin, 2010). The majority of activators were female and as might be expected were younger, fitter, and healthier than the ACE participants.

The qualitative process evaluation indicated that the ACE intervention was well accepted by all parties. ACEs-I adhered to the program, attended the planned sessions with their activators, and in many cases met more often than originally planned. The completion rate was high and only serious health problems and/or family issues prevented some participants from continuing.

This feasibility trial was not powered for statistical significance. However, we observed a positive increase in ACEs-I’ reported activities outside the home. The objective accelerometry data indicated increases in some components of PA for ACEs-C, highlighting the potential for some behavior changes among randomized control trial control group members. However, the low number of follow-up accelerometry data for this group is not sufficient to allow any firm conclusions about PA changes. Activators, although sufficiently active at baseline, also increased their PA supporting the association of volunteering with positive physical health profiles (Anderson et al., 2014). The highest percentage of increased activity for all groups was for lifestyle intensity PA, MVPA, and step counts which points to an increase in out-of-home activity. Although limited, these data support the recent focus on light and lifestyle intensity PA and its importance for older people (Hamer, de Oliveira, & Demakakos, 2014).

Objectively measured physical function increased in 50% of ACEs-I. Similarly, in the LIFE study, the PA intervention group had significantly higher total SPPB scores at 6 months than the control group (Santanasto et al., 2017). This improvement is particularly important as it goes against the expected decline observed in large longitudinal studies. In a sample of 3,070 older adults (mean age = 69), gait speed declined significantly over a 6-year period; evidence of a downward spiral of inactivity and compromised physical function (Shankar et al., 2017). Unlike the LIFE study, ACE was not an intensive exercise intervention, rather indirectly supporting increased PA, but still it produced positive results in terms of physical function. These results also support the supposition that the functional ability of inactive older adults can be very low, and getting out and about for any reason might offer sufficient stimulation for improvement in physical function (Davis et al., 2014; Guzman-Castillo et al., 2017). Combined with these findings, the high prevalence of mobility difficulties in 93% of frail and 58% of non-frail older adults in the UK (Gale, Cooper, & Sayer, 2015), highlight the importance of physical function as a target for public health interventions. Although this study was not powered to detect changes in physical function, it demonstrated the sensitivity of the SPPB measurement tool (Guralnik et al., 1994) in detecting changes in physical function with the target population. The ease of its administration and its objective nature further support its use in future ACE and other active ageing trials.

The quantitative process evaluation supports the applicability of the PMLBC and its usefulness as a theoretical framework for rigorous development and evaluation of such interventions (Gillison et al., 2015). Evidence emerged that focusing on specific behavioral processes in the motivation, action, and maintenance stages and employing strategies aimed at boosting autonomy, competence, and relatedness had been effective. Confidence and social support to get out and about, key determinants of PA participation in older people (Stathi et al., 2012; Stathi, McKenna, & Fox, 2010), increased at follow-up, while the severity of perceived barriers decreased. Together with an increase in resilience, this indicates the potential of the ACE intervention to facilitate lifestyle changes in older people, as these constructs have been shown to promote healthy lifestyles (Manning, Carr, & Kail, 2014; Perna et al., 2012). The positive changes in perceptions of well-being and life satisfaction suggest that the ACE intervention helped participants to improve their quality of life, highlighting the impact of activity on mental health and subjective well-being (Whitehead & Blaxton, 2017).

Any future intervention attempting to build upon this feasibility trial will need to address some specific issues. Recruitment was challenging. This is not surprising, as ACE targeted community-dwelling older adults who were relatively isolated. As such, they are likely to lack the confidence to respond to advertisements regarding new initiatives. Furthermore, we were asking them to pioneer an unfamiliar program that was not yet established in the community. We drew on a wide range of recruitment strategies. Targeted mail/invitations using a commercial database and distribution of flyers locally were the most effective approaches. The response rate was 13% with an overall cost of £2,030 (£9 per response). This rate is comparable to those reported by other community programs such as the Groningen Active Living Model which reported a response rate of 12.5% ($84 per response) (Stevens et al., 2008).

An avenue which was not explored in this feasibility trial was recruitment via primary care (McMurdo et al., 2011). General Practitioners and Practice Nurses have regular contact with older patients and are well placed to identify physical frailty and signs of loneliness or isolation. Social prescribing initiatives could be a promising recruitment strategy for the ACE intervention, offering an opportunity to prescribe non-medical services and holistically support patients with interrelated health and social problems (Kimberlee, 2013). Working closely with community providers of social care, pharmacists and occupational therapists could also assist recruitment. The power of word-of-mouth should also not be underestimated. Further studies of the ACE intervention need to include an internal pilot stage where the feasibility of these recruitment strategies can be established.

A rolling approach to recruitment, providing sufficient time for participants recruited in the early phase to embed themselves in the program, and subsequently share their positive experience with their peers should be tested. This approach is difficult to incorporate within the constraints of a funded research study timeline, as it would extend the duration and hence the costs of a trial. However, research funders have realized the importance and challenges of effective recruitment and given a well-justified rationale, they should be supportive of innovative recruitment models and research designs to enhance trial success. Furthermore, this approach provides a more realistic and pragmatic strategy when implementing and rolling out the program in real world settings.

Participants, particularly in the control group, did not comply well with the instructions about wear time of the waist-worn accelerometers. In a trial with repeated measures, wearing these devices can become a burden. For future trials, we suggest the use of wrist-worn accelerometers which improve compliance rates, as most devices can be continuously worn (Huberty, Ehlers, Kurka, Ainsworth, & Buman, 2015).

The ACE intervention included two social events to which all ACEs-I, activators, and coordinators were invited. These sessions were well received and well attended, and offered further opportunities for meeting new people and exchanging information about local activities. Future studies could facilitate group cohesion in this way and identity other opportunities for interaction among participants. Apart from potentially contributing to improved adherence rates and perceptions of well-being, these events may also support long-term maintenance of positive outcomes for the trial participants.

There was also evidence of organic unplanned growth. Several of the ACEs-I who met during these events formed their own “After ACE” groups, meeting regularly and organizing outings. A future ACE trial will actively support the empowerment of participants to create their own network and become “ambassadors” for the ACE intervention while monitoring the impact of this approach through comprehensive process evaluation. This should contribute to the long-term sustainability and cost-effectiveness of the ACE program.

ACE is a simple and affordable intervention and its potential has been recognized by Public Health England which identified ACE as promising practice in the UK (Public Health England, 2014). It has been adopted by one of the community partners involved in its development and is currently being delivered in the city of Bristol. A full effectiveness and cost-effectiveness pragmatic trial will determine the cost-benefit of delivering ACE.

## Conclusion

This feasibility trial has provided evidence that the ACE intervention is acceptable to all groups of contributors and participants. It is feasible to operate, as costs and labor requirements are relatively low. We have provided preliminary support for the effectiveness of the program in helping isolated and inactive older people to get out and about more, improve their confidence, and engage more with their community. A pragmatic trial employing diverse recruitment strategies, including physical function as one of the key outcomes, promoting the active engagement of participants as ACE Ambassadors and assessing PA with wrist-worn accelerometers will provide definitive evidence about the effectiveness and cost-effectiveness of this promising intervention.

## Funding

This study was supported by Medical Research Council - Lifelong Health and Wellbeing (grant/award number: G1001864).

## Conflict of Interest

None reported.

## Supplementary Material

gnz003_suppl_Supplementary_Material
