# Supplementary material for: Feasibility Trial Evaluation of a Peer Volunteering Active Aging Intervention: ACE (Active, Connected, Engaged)
Source: Gerontologist. 2019 Feb 19;60(3):571–82. doi: 10.1093/geront/gnz003 (PMC7117619; doi:10.1093/geront/gnz003)
Supplement: gnz003_suppl_Supplementary_Material [file gnz003_suppl_supplementary_material.docx]

Appendix A

Suggestions for ACE improvements

| **Areas for improvement** | **Feedback (bold) and Quotations (italics)** |
| --- | --- |
| Activators training | **Take into account the wide range of activators’ skills and experience ensuring that the information provided is not overwhelming:** *I have learnt so much, I wouldn’t have been so easy going into someone’s house, it has helped me with that. I feel so much easier going into someone’s house and talking with them. (Activator, Group 1)*  *Yes, I did find it useful. The only thing I might say against it, it was a bit overwhelming at the start, because you think ‘this is a bit more complicated than I thought’. But on reflection, you’ve got to give all the information, and you just pick out the bits that you need, I suppose. (Activator, Group 1)*  *I think when you got a group of very mixed abilities, you’ve always got to pitch the activities and the whole sort of agenda for the day at the lowest denomination. (Activator, Group 2)* |
| Activators’ on-going support | **a) Receive more information about the participants before first meeting**  **b) More information about potential activities:** *Maybe more information, not sort of factual about the person you’re meeting with, but their capabilities, and that would help you where to go, which direction perhaps, in what you’re going to have to deal with, rather than just a name and a little bit of information. (Activator, Group 1)*  **c) Engage activators to actively identify suitable activities:** *If you had given us …a more specific exercise to find things, we would have got more out of it. Maybe during the second training day?(* *Activator, Group 2)* |
| Recruitment | **Recruitment via trusted sources:** *Maybe people’s doctors could tell them about it because then people trust ….you know …if it was coming from someone they trusted.* (ACE participant, Group 2)  **Recruitment via health centres and libraries where professional staff tend to know potentially eligible participants well :** *I mean, I think libraries, health centres, doctor surgeries, ACE neighbours, where somebody working there has a slight idea of what you’re doing, and could possibly help slightly promote it*. (ACE participant, Group 2)  **Word of mouth:** *I think the most effective way of doing it, is word of mouth, and having an example, so, it’s then reaching those people who don’t go anywhere. They’re likely to listen to the radio, maybe read a newspaper and watch the TV* (Activator, Group 2) |
| Assessment  Questionnaires | **Continue using an interviewer-administered questionnaire approach:** *I think being measured up and all that wasn’t a problem. We had someone there filling in the form for us at the end, pages and pages of it.*  (ACE participant, Group 2)  **Use simpler wording:** *That was fine, there were a few strange ones, the language sometimes, I had to think, and read out the questions ‘Now, what am I answering?’ So, again, it was the language sometimes.* (Activator, Group 2) |
| Contact forms | **Simplify the process of filling in forms in every meeting with ACEs:** *It was cumbersome really. It could be an easier way, like a sort of diary entry, rather than having to fill in that form every time you did a phone call. (ACE participant,, Group 1)*  *I wasn’t very happy filling in forms, I didn’t do it in the end. I left that in the end because you sit there talking to someone and you say I’m sorry I’ve just got to fill this in… they don’t want that. (Activator, Group 1)* |
